# Study on the Electrical Insulation Properties of Modified PTFE at High Temperatures

**DOI:** 10.3390/polym16030316

**Published:** 2024-01-24

**Authors:** Lijian Yuan, Xu Zheng, Wenbo Zhu, Bin Wang, Yuanyuan Chen, Yunqi Xing

**Affiliations:** 1 School of Electrical and Electronic Engineering, Shandong University of Technology, Zibo 255000, China; ylj_2006@163.com; 2 Shandong Dianliangliang Energy Technology Co., Ltd., Jinan 250000, China; 3 State Key Laboratory of Reliability and Intelligence of Electrical Equipment, Hebei University of Technology, Tianjin 300130, China; zhengxu_0224@163.com (X.Z.); w604117@163.com (B.W.); yychebut@163.com (Y.C.); 4 China Southern Power Grid Electric Power Research Institute, Guangzhou 510000, China; zhuwb@csg.cn

**Keywords:** aviation electrical insulation, polytetrafluoroethylene, magnetron sputtering, surface flashover, electric aging

## Abstract

During the operation of multi-electric aircraft, the polytetrafluoroethylene (PTFE) material used to insulate the aviation cable is subjected to a high electric field while working under the extreme conditions of high temperatures for a long time, which can easily cause a partial discharge and even flashover along the surface, which seriously threaten the safe operation of the aircraft. In this paper, the electrical insulation properties of PTFE were regulated via modification by the magnetron sputtering of TiO_2_ under high temperatures, and modified PTFE with different sputtering times was prepared. The direct current (DC) surface discharge, surface flashover, and electric aging characteristics of modified PTFE were studied under the condition of 20~200 °C, and the mechanisms by which modification by sputtering of TiO_2_ and high temperature influence the insulation properties were analyzed. The results show that the surface discharge intensity increases with the increase in temperature, the modification by sputtering of TiO_2_ can significantly inhibit the partial discharge of PTFE, and the flashover voltage first increases and then decreases with the increase in the modification time. The modification by magnetron sputtering can effectively increase the surface potential decay rate of the PTFE, increase the shallow trap energy density, effectively avoid charge accumulation, inhibit the partial discharge phenomenon, and improve the surface electrical insulation and anti-aging properties.

## 1. Introduction

The air transport industry is bringing about more and more serious problems, such as carbon emissions and noise pollution. However, it is difficult to reduce carbon emissions with an internal combustion engine that uses fossil fuel. So, innovations in the energy system and improvements in the efficiency of energy use in order to reduce greenhouse gas emissions are needed. In addition, in traditional aircraft, hydraulic and pneumatic systems require a large number of pipelines to perform their functions, and they occupy a large amount of space inside the aircraft. Their cumbersome operating mechanism makes the system work less efficiently. Meanwhile, the complicated operation and high failure rate reduce the operational reliability of the aircraft [1,2,3,4].

The arrival of the electrical age ushered in large-scale electrification to aircraft control systems. Hydraulic and pneumatic systems with relatively low energy efficiency and a complex mechanical structure were replaced, and the structure of the aircraft’s energy system was optimized. Thus, the concept of a multi-electric aircraft was born, which subverted the design formula of a traditional aircraft’s secondary energy system, consisting of a hydraulic system, a pneumatic system, and an electrical system [5,6,7]. The secondary energy system of a multi-electric aircraft employs more electrified equipment, which can reduce the types of energy supply systems on the aircraft, greatly reduce the complexity of these systems, and improve the reliability of the aircraft. At the same time, the energy efficiency of electrified equipment is much higher than that of hydraulic and pneumatic systems, which can effectively improve the fuel efficiency of the aircraft and reduce carbon emissions [8,9,10]. Multi-electric aircraft technology has been applied in various types of aircraft, such as the A380, the B787, and the F-35, and has shown great value and development potential in the application process [3,11].

The reliability of the insulation of an aviation cable is an important factor in ensuring the safe and stable operation of aircraft. The high current passing through the cable during operation will lead to high working temperatures [12] of up to about 200 °C [13,14]. These working temperatures will seriously age the insulation materials, can easily induce partial discharge and flashover accidents, and seriously threaten the safe operation of the aircraft [15]. With the development of multi-electric aircraft, the bus voltage level will be further improved, the insulating material of the aviation cable will withstand a stronger electric field, and the accumulation of more electric charges on the surface of the insulating material will further aggravate the electric field distortion. Therefore, the surface insulation characteristics and electrical aging characteristics of aviation cable materials under extreme conditions are key issues that need to be studied. Fluoropolymer materials, such as PTFE, can greatly improve the insulation performance of cables. So, they are widely used in aviation cable insulation materials. PTFE, which is polymerized from a tetrafluoroethylene monomer, has a highly symmetrical molecular structure and its carbon chains are connected with high-energy C–F bonds, which provide it with excellent physical and chemical properties [16,17]. However, there remain certain deficiencies in the current insulation performance of PTFE in extreme aviation environments. In order to improve the insulation performance of aviation cables, with the exception of adopting insulation materials with better performance, the existing insulation materials are usually modified in various ways [18].

Guo et al. observed the influence of the surface roughness of the PTFE material on the luminescence phenomenon and discharge path before surface flashover using enhanced charge-coupled devices. They found that PTFE material with a rough surface can effectively suppress the accumulation of surface charge, and the probability of a discharge path appearing in the rough surface area is much lower than that on a smooth surface area [19]. Yanqing et al. found that the deep trap content of micro–nano Al_2_O_3_-modified PTFE increased significantly, and the DC breakdown voltage also increased significantly [20]. Dongxin et al. studied the electrical aging characteristics of PTFE materials under the combined effects of electrical heating, and observed the surface after electrical aging treatment with infrared spectroscopy and scanning electron microscopy (SEM). They found that, as the electrical aging time increased, many holes and cracks appeared on the surface of the PTFE [21]. Duan et al. carried out a surface fluorination treatment of polyethylene materials. The fluorinated layer generated by the surface modification increased the surface roughness of the insulating materials, suppressed the secondary electron emission process, and thus increased the surface flashover voltage under vacuum conditions [22]. It can be seen that current research on the modification of PTFE materials mainly focuses on surface charge accumulation and dissipation, as well as flashover and breakdown characteristics. There are few studies on the modification by surface sputtering of PTFE materials, intended to suppress discharge and improve resistance to electrothermal aging.

In recent years, scholars have found that, compared with modification by doping, surface modification by magnetron sputtering can further increase the surface roughness of insulating materials, and thus promote the dissipation of surface charge, and this has a more significant improvement effect on the surface insulation of insulating materials [23]. It also has the advantages of a high deposition rate and high quality assurance of the film formed, and has been widely used in improving the surface properties of insulating materials [24]. Current studies have shown that titanium oxide (TiO_2_) has an excellent chemical stability and good mechanical properties. As a semiconductor material, it also has a low trap energy level and density, which struggles with the accumulation of electric space charges, and can accelerate the electric charge dissipation of the material. TiO_2_ can be used to modify insulating materials, which can effectively reduce the degree of electric field distortion on the surface of the material and improve its surface insulation performance [25,26,27].

Therefore, in this paper, magnetron sputtering with TiO_2_ was used to regulate the insulation properties of PTFE materials, the surface insulation properties and electrical aging characteristics were studied, and the influence mechanisms of the modification of magnetron sputtering with TiO_2_ on the surface flashover and electrical aging process were analyzed.

## 2. Materials and Methods

### 2.1. Materials Preparation

**Figure 1:**
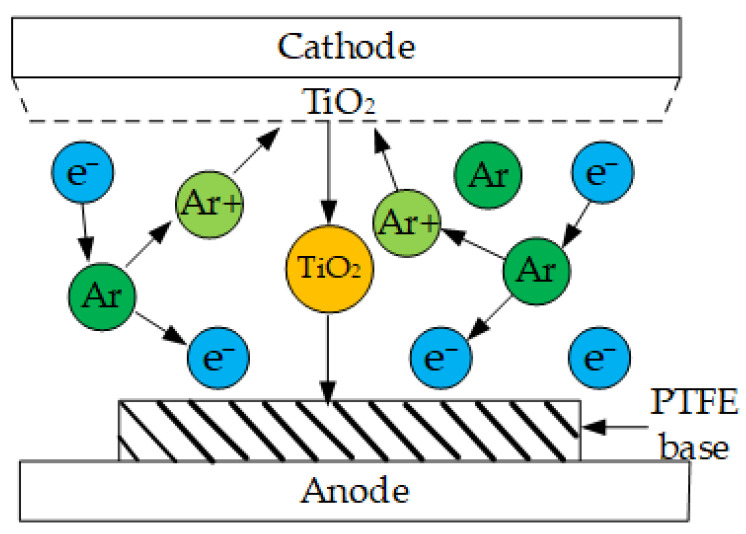
Working principle of magnetron sputtering.

The PTFE material used in this paper was a plate produced by Shanghai Jianzuo Industrial Co., Ltd., Shanghai, China. The CAS number is 9002-84-0, and the manufacturing method was combined cold-pressing and sintering. The shape and size of the PTFE were selected as a disc with a diameter of 130 mm and a thickness of 2 mm. The TiO_2_ material was an analytical pure powder produced by Tianjin Zhiyuan Chemical Reagent Co., Ltd., Tianjin, China. Its CAS number is 13463-67-7, and the manufacturing method was sulfuric acid hydrolysis and precipitation roasting. First, the TiO_2_ target and the PTFE substrate were cleaned by application of a stream of pure nitrogen gas to remove impurity particles from the surface. Then, they were placed on both sides of the cavity to serve as the cathode and anode, respectively. Vacuum treatment was performed in the cavity of the magnetron sputtering instrument to maintain the air pressure at 3 × 10^−3^ Pa. Before magnetron sputtering, argon gas was injected into the cavity at a volume flow rate of 40 sccm until the air pressure in the cavity reached 0.5~1 Pa, and the RF power was set to 200 W to prevent substrate deformation caused by excessive temperatures in the cavity. Through the acceleration of the external electric field, electrons were collided with argon atoms in the cavity at a very high speed, resulting in an argon-positive ion and a secondary electron. The argon-positive ion carried high kinetic energy and bombarded the TiO_2_ target, causing TiO_2_ molecules on the target to break away from the surface for sputtering. These sputtered TiO_2_ molecules attached to the PTFE substrate and precipitated to form a dense TiO_2_ coating. In this study, the modification times of magnetron sputtering were selected as 0, 15, 30, 45, and 60 min, and a total of five modified samples were prepared. The schematic diagram of the process of modification by magnetron sputtering is shown in Figure 1.

### 2.2. Experimental Method

**Figure 2:**
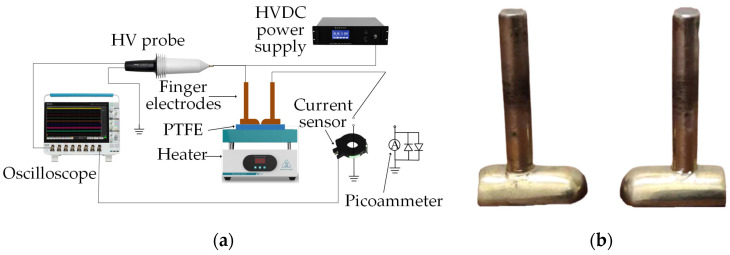
High-temperature surface discharge and electrical aging test platform. (**a**) Overall arrangement; (**b**) enlarged diagram of the electrodes.

The high-temperature surface discharge and electrical aging test platform built for the experiment is shown in Figure 2. Figure 2a shows the overall arrangement, and Figure 2b is an enlarged diagram of the electrodes. The model of the high-voltage (HV) DC power supply used in the experiment was a DW-P603-3ADC2, produced by TianjinDongwen High Voltage Power Supply Corp., Tianjin, China, the purpose of which is to create an electric potential between the electrodes. The HV probe model was TT-HVP2739, the objective of which is to measure the voltage of the electrode. The current sensor model was an HFCT-1607022, produced by TESTEC Elektronik GmbH, Dreieich, Germany, the objective of which is to measure the discharge current. The oscilloscope model was MSO54, produced by Tektronix Inc., Beaverton, OR, USA, the objective of which is to measure the waveform of the voltage and current. And the picoammeter was a Keithley6514 model, produced by Tektronix Inc., Beaverton, OR, USA, the objective of which is to measure the leakage current. The electrode used in the experiment was a brass finger electrode, which is composed of a semi-cylinder with a radius of 4 mm and a 1/4 sphere with a radius of 4 mm, and the distance between the electrodes was fixed at 8 mm. A heater was placed under the PTFE sample to simulate conditions wherein the sample is heated by the electrical current passing through the cable. In the test environment, the relative humidity was kept constant at 30% and the air pressure was one standard atmosphere. Before the experiment, the samples were first cleaned, then dried and discharged. Then, the test temperatures were applied and maintained with the heater at 20, 65, 110, 155, and 200 °C, respectively. During the discharge test, one of the finger electrodes was grounded through the current sensor, and the voltage was increased at the rate of 0.1 kV/s until a flashover occurred. The initial partial discharge voltage and the surface flashover voltage during the flashover process were recorded. Then, the voltage was increased to the initial partial discharge voltage at the same rate, and the average discharge amplitude and discharge repetition rate of the sample were recorded, respectively. During the electrical aging test, the voltage was increased at the rate of 0.1 kV/s until a flashover occurred and the voltage remained unchanged for 4 min to complete the electrical aging process. We then reduced the voltage, discharged and switched the finger electrode to ground through the picoammeter, and applied 2 kV DC voltage. An upper computer program connected with the picoammeter was used to measure the surface leakage current, and the reading was recorded after the leakage current value had stabilized. Then the sample was taken out. The mass loss of the sample was measured via the JJ224BC precision analytical balance, produced by G&G Measurement Plant, Changshou, China, and the water contact angle of the sample was measured using a DSAHT drop shape analysis system.

In order to better observe the effect of magnetron sputtering on the original, modified, and electric aged PTFEs, SEM tests were conducted in this paper. In order to verify the chemical composition of the PTFE surface-modified by magnetron sputtering, infrared spectral absorption was also tested, and the equipment model was BRUKER VERTEX 80V2, produced by Bruker Corporation, Billerica, MA, USA. The DC surface conductivity of the modified PTFE material was tested using the three-electrodes method. The wiring and experimental method were those set out by the China National Standard GB/T31838.2-2019 [28].

## 3. Results

### 3.1. Sputtering Modification Characteristics

**Figure 3:**
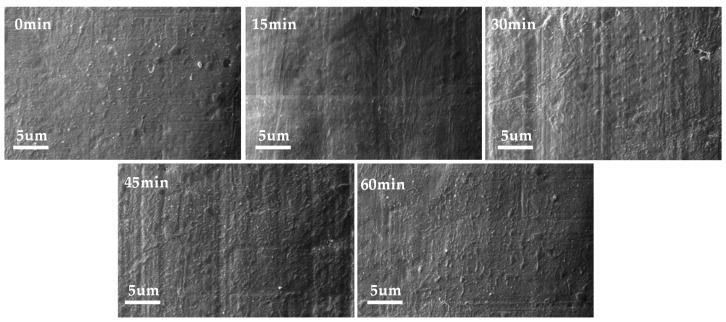
SEM of modified PTFE.

The results of SEM tests on the original and modified PTFEs are shown in Figure 3. As can be seen from the figure, the surface of the original PTFE sample without modification by magnetron sputtering was relatively smooth, and only granular processes appeared in a small part of the area. As the sputtering time increased to 15 min, the thickness of the deposited layer on the surface of the PTFE increased, and the TiO_2_ coating left by magnetron sputtering uniformly covered the surface of the PTFE. Defects, such as uneven ups and downs on the surface of the original sample, were covered by the dense film forms. As the modification time of magnetron sputtering further increased to 45 min, fine striated gullies appeared on the surface of the sample with a more consistent and uniform direction and distribution, which was caused by the temperatures rising in the cavity and the slight shrinkage of the surface coating during the magnetron sputtering process. Compared with the sample made with shorter sputtering times, most of the fine grooves on the surface of the sample with a modification time of 60 min disappeared, but it was still rougher than the original PTFE. On the whole, the TiO_2_ films on the surface of the PTFE samples modified by magnetron sputtering were uniform and dense, and there was no large-area accumulation phenomenon.

**Figure 4:**
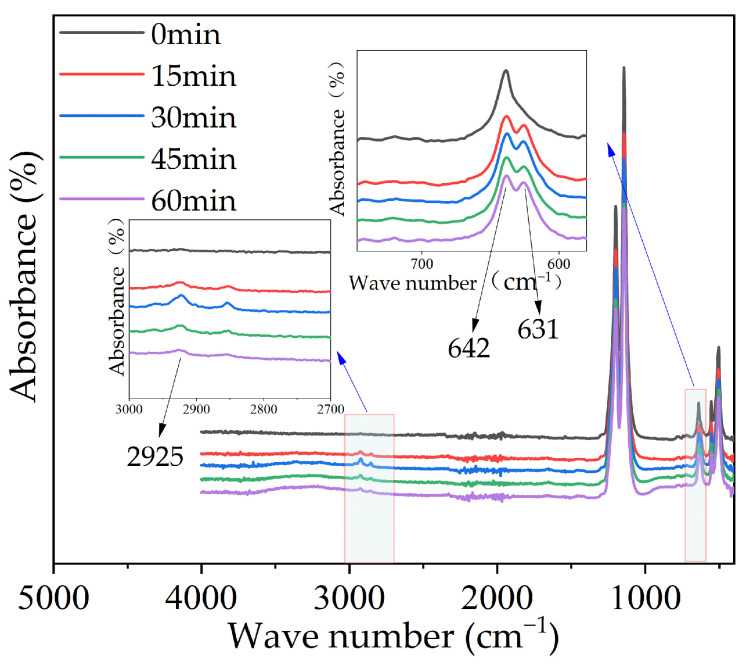
Infrared spectral absorptions of modified PTFE.

The infrared spectral absorptions of the original and modified PTFE are shown in Figure 4. As can be seen from the figure, in addition to the changes in the strength of the peak of the F–C–F bond, new peaks were generated at wave numbers of 631 and 2925 cm^−1^, respectively, corresponding to the variable angle stretching vibration of the O–Ti–O bond and the stretching vibration of the Ti–O bond, indicating that the TiO_2_ film was successfully formed on the surface of the PTFE by magnetron sputtering.

### 3.2. Surface Conductivity Characteristics

**Figure 5:**
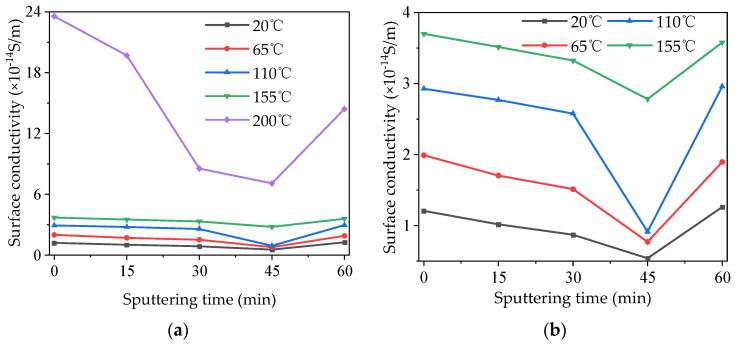
Surface conductivity of modified PTFE at different temperatures. (**a**) All temperature conditions; (**b**) the details at lower temperatures.

The DC surface conductivity of the modified PTFE was tested at high temperatures, and the test results are shown in Figure 5a. Because the surface conductivity at 200 °C was much higher than that at other temperatures, the surface conductivity at other temperatures was extracted and turned into its own figure, Figure 5b, to present its characteristics more clearly. As can be seen from Figure 5a,b, under each temperature condition, the surface conductivity of the samples first decreased and then increased with the increase in the modification time of magnetron sputtering. The surface conductivity of samples at 0 min was the highest, while that of samples at 45 min was the lowest. In the range of 20~155 °C, the surface conductivity of the samples was lower than 4 × 10^−14^ S/m, and it increased with the increase in temperatures. When the temperature reached 200 °C, the surface conductivity of modified PTFE significantly increased, and the surface conductivity of the 0 min sample at 200 °C increased by 15 times compared with that at 20 °C. When the temperature was 200 °C, the correlation between the surface conductivity of modified PTFE and the modification times of magnetron sputtering was significantly enhanced, and the surface conductivity of the 45 min sample decreased by 70.83% compared with that of the 0 min sample. In conclusion, TiO_2_ modification by magnetron sputtering can effectively regulate the surface conductivity of the PTFE, and the regulation effect is more significant under high-temperature conditions.

### 3.3. Initial Characteristics of Surface Discharge

**Figure 6:**
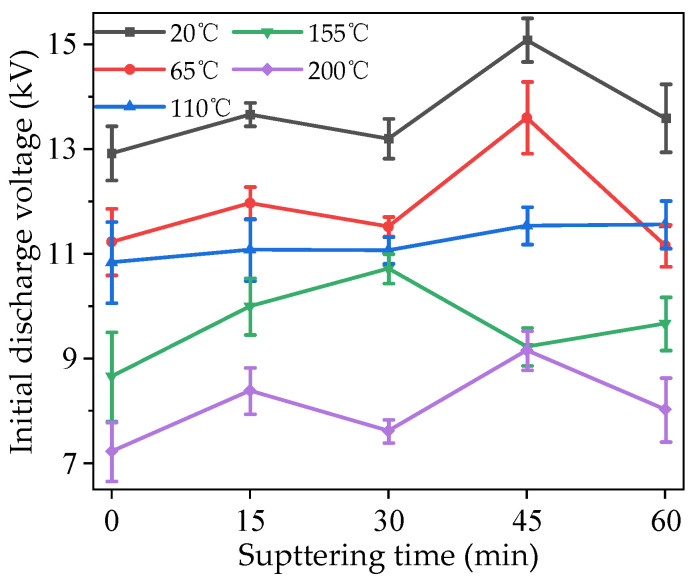
Initial discharge voltage of modified PTFE at different temperatures.

The modified PTFE was tested 10 times in each group, and the experimental data were processed via their Weibull distribution. The corresponding value of 63.2% probability was selected for analysis. The initial discharge voltages of the modified PTFE under different temperature conditions are shown in Figure 6.

It can be seen from the figure that, for samples with the same sputtering times, the initial voltage of surface discharge decreased to different degrees with the increase in temperature. The initial discharge voltage of the samples without sputtering decreased by 1.69 and 2.16 kV, respectively, between 20 and 65 °C and 110 and 155 °C, accounting for 13.09% and 16.73% of the voltage at 20 °C, respectively. After 15 min sputtering, the initial discharge voltage distribution of the sample was more uniform than that of the sample without sputtering. When the sputtering time was 30 min, the initial discharge voltages at 65, 110, and 155 °C were relatively close, and the differences were only 0.45 and 0.34 kV, accounting for 3.41% and 2.58% of the voltage at 20 °C, respectively, but this difference is larger than those at 20 and 200 °C. When the sputtering time was 45 min, the initial voltages of surface discharge at 155 and 200 °C were close to each other, with a difference of only 85 V, but this difference is larger than that at other temperatures. When the sputtering time reached 60 min, the initial surface discharge voltage at 65 °C was lower than that at 110 °C, with values of 11.16 and 11.53 kV, respectively, indicating that sputtering for too long may reduce the insulation performance of the PTFE at certain temperatures. Under the same temperature conditions, with the increase in sputtering times, the initial voltages of surface discharge first increased and then decreased. The initial discharge voltage increased after 15 min of sputtering at all temperatures. When the temperature was 20, 65, and 200 °C, the changes in initial discharge voltages were basically the same. All of them decreased at 30 min, increased at 45 min to the maximum value, and decreased again at 60 min. When the temperature was 110 °C, the initial voltage of the surface discharge increased monotonically with the increase in sputtering time, and the amplitude of each increase was small. When the temperature was 155 °C, the maximum initial voltage of surface discharge appeared on the sample whose sputtering time was 30 min. It can be seen that modification by magnetron sputtering can significantly improve the insulation properties of PTFE, but the control effect is related to the sputtering time and working temperatures.

### 3.4. Development Characteristics of Surface Discharge

**Figure 7:**
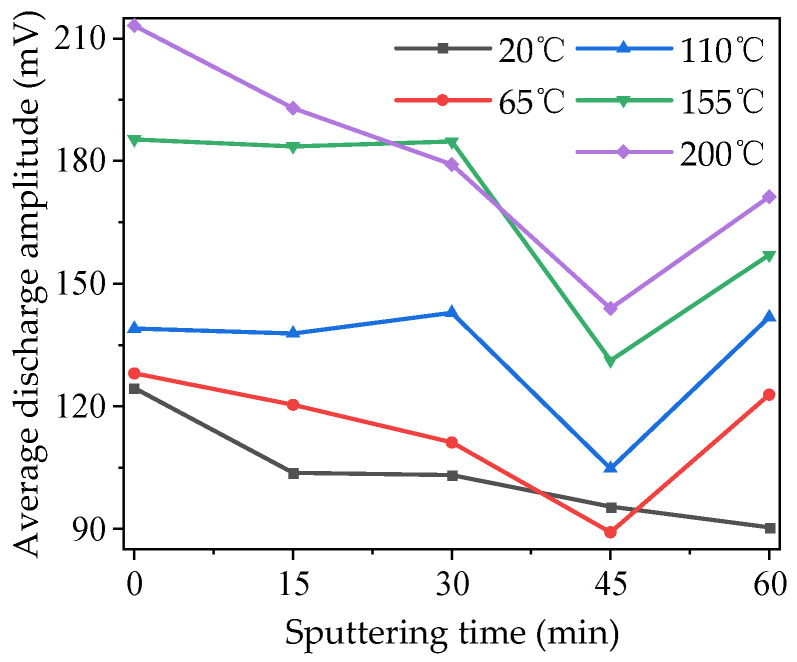
Average discharge amplitude of modified PTFE at different temperatures.

The average discharge amplitude of modified PTFE under different temperature conditions is shown in Figure 7. It can be seen that with the increase in temperatures, the average discharge amplitude of each sample increased to different degrees on the whole, and the gap between 110 and 155 °C was the largest. With the increase in sputtering time, the average discharge amplitudes of samples at different temperatures first decreased and then increased. The variation trends of the average discharge amplitudes of samples at 15 and 30 min were significantly different at different temperatures. However, the average discharge amplitude of the 45 min sample decreased significantly at all temperatures except 20 °C, and the average discharge amplitude of the sample at 200 °C decreased the most—it was 32.9% lower than that of the unmodified sample. It can be seen that modification by magnetron sputtering can significantly inhibit the development of a partial discharge. When the sputtering time further increased to 60 min, the average discharge amplitude of the sample under other temperature conditions except 20 °C showed a significant rebound. And the average discharge amplitude at 110 °C exceeded the value without modification, which means the modification by magnetron sputtering had a negative effect.

**Figure 8:**
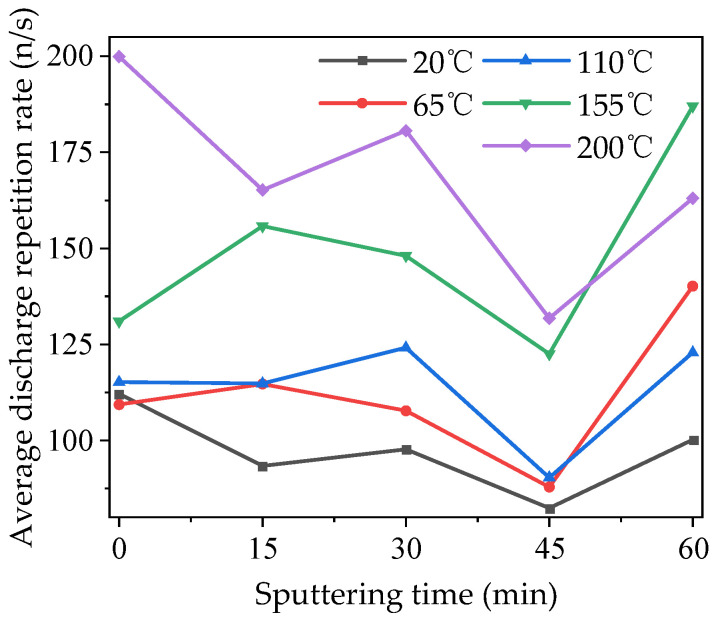
Average discharge repetition rate of modified PTFE at different temperatures.

The average discharge repetition rate of modified PTFE at different temperatures is shown in Figure 8. As can be seen from the figure, similar to the characteristics of the average discharge amplitude, the average discharge repetition rate of each sample also increased to different degrees on the whole with the increase in temperature, and the largest gap appeared between 110 and 155 °C. Compared with the average discharge amplitude, the average discharge repetition rate of samples at different temperatures fluctuated more, and the regularity was worse when the sputtering time was 15 min and 30 min. However, when the sputtering time increased to 45 and 60 min, the average discharge repetition rate at all temperatures showed the same characteristics as the average discharge amplitude, which decreased significantly at 45 min and rebounded at 60 min. It can be seen that modification by magnetron sputtering can also inhibit the probability of a partial discharge.

### 3.5. Surface Flashover Characteristics

**Figure 9:**
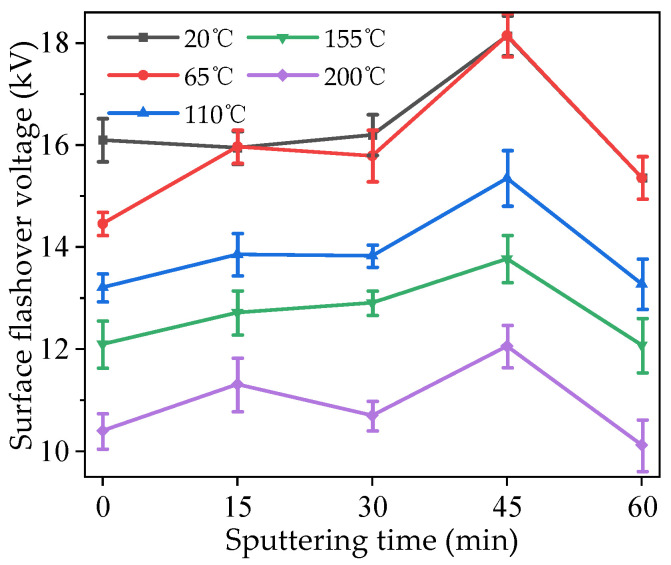
Surface flashover voltage of modified PTFE at different temperatures.

The surface flashover voltage of modified PTFE at different temperatures is shown in Figure 9. It can be seen in the figure that, for samples with the same sputtering times, when the temperature was low, some of the surface flashover voltage values of 20 and 65 °C were very close or coincided, indicating that the temperature rise in the lower temperature range had little effect on the insulation performance of modified PTFE. With the further increase in temperature, the surface flashover voltage decreased obviously, and the higher the temperatures, the lower the surface flashover voltage. When the temperature reached 200 °C, the drop in surface flashover voltage was greater than that seen with the previous temperature rise, indicating that the high-temperature environment has a great influence on the insulation performance of modified PTFE. Under the same temperature condition, with the increase in sputtering time, the surface flashover voltage first increased and then decreased. The surface flashover voltage increased after 15 min sputtering except in the 20 °C conditions. When the sputtering time increased to 30 min, the surface flashover voltage under different temperature conditions increased and then decreased compared with 15 min, and the regularity was not obvious. When the sputtering time was 45 min, the surface flashover voltage values were the highest for each sputtering time, and the insulation performance of the modified PTFE was the best. When the sputtering time further increased to 60 min, the surface flashover voltage values significantly decreased, indicating that a longer the sputtering time did not necessarily correlate with a better insulation performance of the modified PTFE.

### 3.6. Electric Aging Characteristic

**Figure 10:**
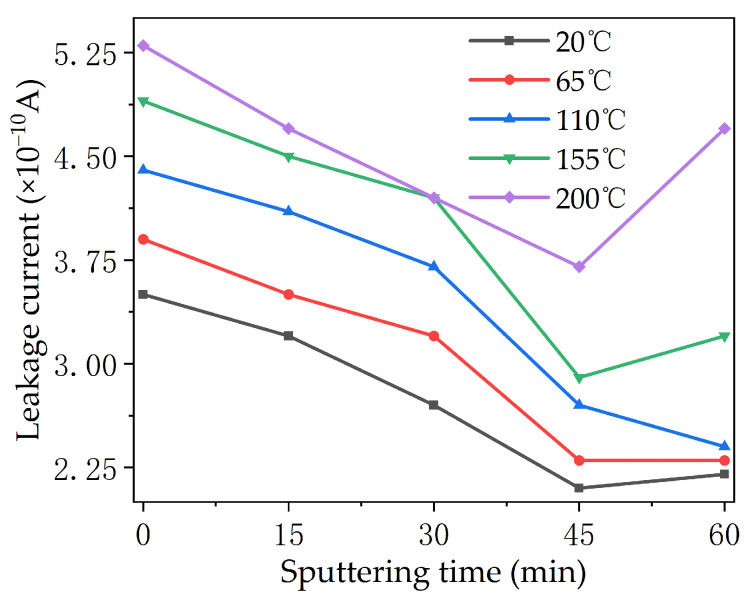
Surface leakage current of modified PTFE after electric aging at different temperatures.

Figure 10 shows the leakage current measured from the PTFE after the electrical aging test. The leakage current on the surface of each sample increased with the increase in temperatures. Except for the samples at 110 °C, the leakage current of the samples at other temperature conditions showed a trend of first decreasing and then increasing with the increase in sputtering times. On the whole, the surface leakage current of the 45 min sample was the smallest under all temperature conditions, and the surface leakage current of the 45 min sample at 20 °C reached as low as 2.05 × 10^−10^ A, which is much lower than those of other modified samples. The surface leakage current of the sample sputtered for 60 min at 200 °C showed an abnormal and large increase, indicating that the insulation performance of the PTFE may be reduced after a long period of sputtering.

**Figure 11:**
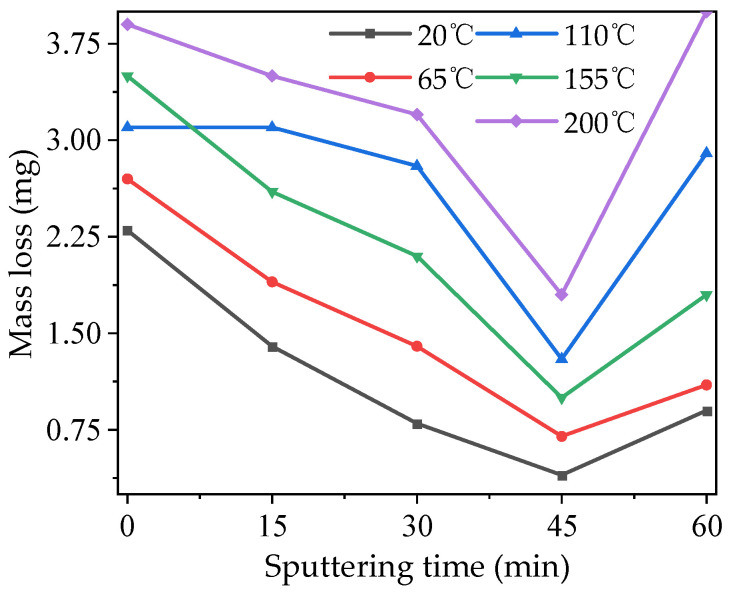
Mass loss of modified PTFE after electric aging at different temperatures.

The mass loss of modified PTFE after electric aging is shown in Figure 11. As can be seen from the figure, the mass loss of modified PTFE first decreased and then increased with the increase in the modification time of magnetron sputtering TiO_2_ under each temperature condition, and the sample with a modification time of 45 min showed the lowest mass loss under each temperature condition. At the same time, temperature was also an important factor affecting the electric aging characteristic of modified PTFE. The mass loss of modified PTFE showed an increasing trend with the increase in temperatures, and the influence of sputtering time on mass loss was more obvious when the temperatures were higher. The above phenomenon shows that the modification of TiO_2_ by magnetron sputtering can effectively improve the anti-electric-aging ability of the PTFE, but the appropriate modification time should be selected. Further, modification for too long under high-temperature conditions makes the anti-electric-aging ability of the modified PTFE even weaker than that of the original PTFE.

**Figure 12:**
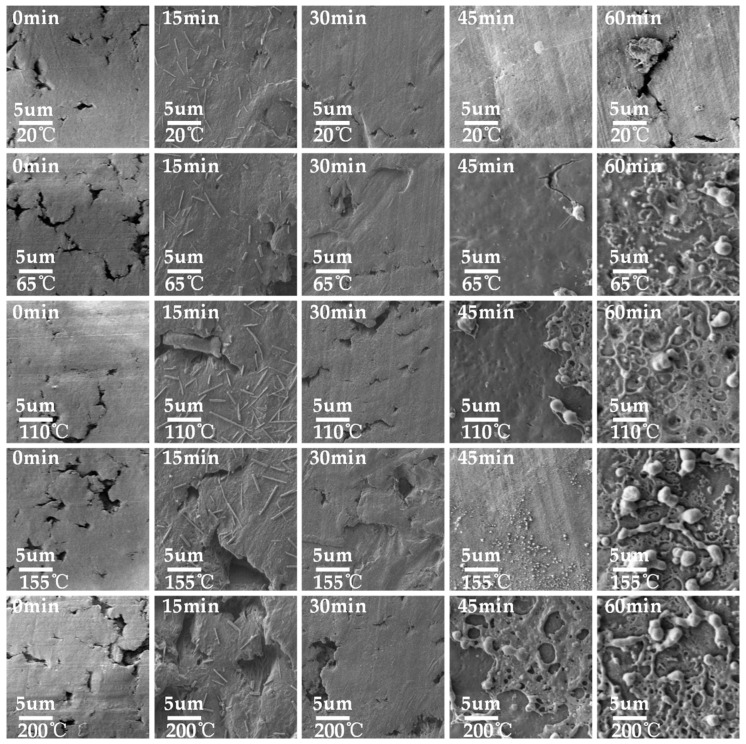
SEM of modified PTFE after electric aging at different temperatures.

The results of SEM tests on electric-aged PTFE are shown in Figure 12. Under the condition of 20 °C, large holes and cracks appeared in most areas of the surface of the sample at 0 min, and fibrous adhesion mostly occurred in the cracks, which was mainly caused by the large amount of heat released during the discharge process. Compared with the sample at 0 min, the surface damage of the 15 and 30 min samples was still large, and there were deep electric marks around the damage of the holes. There were cracks and holes on the surface of the 45 min sample, but the distribution area was small and the depth as shallow. Within 60 min, the pores on the surface of the sample were seriously damaged, and the surrounding areas showed aging and cracking. At 65 °C, the degrees of surface damage of the 0 and 15 min samples were similar, and deep electric marks were generated on the surfaces of the material due to partial discharges, with cracks overlapping in the whole region. Although the surface of the 30 min sample also showed hole damage under electric aging, the damage area was smaller and shallower, and no obvious electric trace damage occurs. The surface hole damage of the 45 min sample was smaller and shallower, and there was no obvious electric trace damage. At 110 °C, a flocculent film appeared on the surface of the 45 min sample, and the area below the film was smooth and showed no obvious damage. At the surface crack of the 60 min sample, there were some filamentous material connections, and the surface of the material showed decomposition and aging. With the increase in the experimental temperature, the surfaces of the 0, 15, and 30 min samples decomposed, and there were fine filamentous fibers attached to the surfaces of the samples, while the sample of 45 min only showed a small amount of superficial damage due to the protection of the TiO_2_ film with the appropriate thickness. At 200 °C, small particles of TiO_2_ film produced by sputtering modification on the surface were attached to the surface of the sample due to high-temperature degradation, such that the sample at 45 min was less affected by electric aging. Meanwhile, due to the short sputtering times, the thicknesses of TiO_2_ films on the surfaces of the 15 and 30 min samples were not enough to protect the PTFE. Under the action of long-term electric aging, the TiO_2_ film on the surface was damaged and produced mesh holes, leaving the PTFE directly exposed to the action of electric discharges, and also leading to deep hole damage.

**Figure 13:**
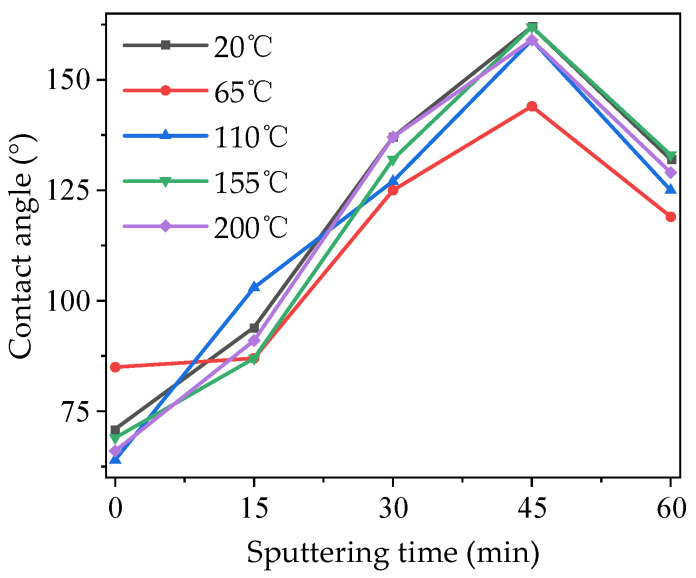
Contact angle of modified PTFE after electric aging at different temperatures.

The surface water contact angles of electric-aged PTFEs were tested, and the results are shown in Figure 13. As can be seen from the figure, the water contact angle of each sample first increased and then decreased with the increase in sputtering time, and it reached its maximum when the sputtering time was 45 min, at which time the sample showed strong hydrophobicity. Then, all of the samples’ angles decreased when the sputtering time was 60 min. The contact angles of all samples at 0 min and some of the samples at 15 min after electric aging were less than 90 degrees, and still showed slight hydrophilicity. The influences of different temperature conditions on the contact angles of the PTFE after electric aging were not obvious, and the increases in temperature only increased the contact angle characteristic curves slightly.

## 4. Discussion

### 4.1. Potential Decay Characteristic

**Figure 14:**
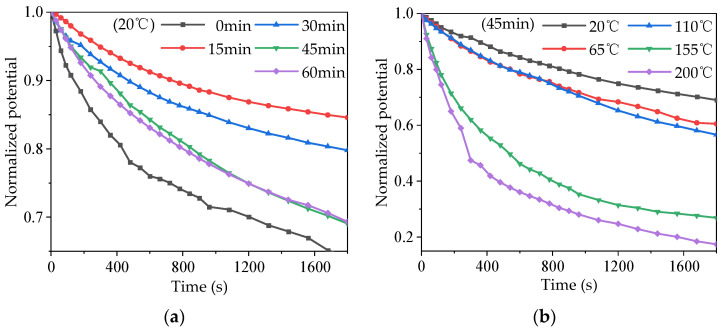
Surface electric charge attenuation characteristics of the PTFE. (**a**) Different modification times at 20 °C; (**b**) different temperatures at 45 min.

In order to study the effects of different modification times and temperatures on the surface electric charge movement of the PTFE, isothermal surface potential attenuation tests were carried out at 20 °C with different modification times, and for 45 min at different temperatures, respectively. The experiment device comprised needle–gate combined electrodes. Firstly, a voltage of ±6 kV was applied to the needle electrode, and a voltage of ±3 kV was applied to the gate electrode, and the PTFE sample was corona charged for 20 min. The sample was then quickly moved under the Kelvin probe to measure the surface potential attenuation characteristics, and the test duration was 30 min. Due to the difference in the surface potential after corona charging, the surface charge is normalized by dividing the surface potential at a certain time by the initial potential. The surface charge decay curves of the PTFE at different modification times and temperatures are shown in Figure 14.

It can be seen from Figure 14a that the decay rate of the surface electric charge of the unmodified sample was the fastest at 20 °C, and when the decay time was 1800 s, the remaining surface charge was only 64.2%. When the modification began, the charge decay rate of the sample surface decreased to the slowest rate, and then accelerated gradually with the increase in modification time. The charge decay rate of the 15 min sample was the slowest, with 84.6% of the surface charge remaining at the time of 1800 s. After 1000 s, the surface charge decay rates of the samples with the modification time of 45 and 60 min were basically the same. As can be seen from Figure 14b, for the sample modified for 45 min, the higher the temperature, the faster the surface charge decay rate. The remaining surface charge was 69.1% at 1800 s at room temperature (20 °C). When the temperature was 65 and 110 °C, the charge residual rate before 700 s was basically the same. When the temperature increased to 155 °C, the decay rate of surface charge increased significantly compared with the previous temperatures. At the temperature of 200 °C, only 17.4% of the surface charge remained at 1800 s.

### 4.2. Trap Distribution Characteristic

In the corona charging process during the surface potential test, the positive corona charging injected holes into the surface of the material, while the negative corona charging injected electrons into the surface of the material. After the corona charging process, the surface potential of the material entered a decay process, in which the electrons or holes on the surface of the material flowed into the earth through the ground electrode. In order to further analyze the effects of modification by magnetron sputtering on the surface electric charge behavior of the PTFE, according to the surface potential attenuation theory, the trap distribution characteristics of the material surface can be obtained using Formula (1) [29].

(1) $$N_{t}(E)=E_{t}\frac{4\varepsilon_{0}\varepsilon_{r}\;}{eL^{2}k^{2}T^{2}\ln(\nu t)}\left|\;t\frac{dV(t)}{dt}\right|$$

The meanings of the variables in Formula (1) are as follows: *E_t_*—energy level of the sample trap/eV, *N_t_*—density at the corresponding energy level/(eV^−1^·m^−3^), (ε_0_ε_r_)—dielectric constant of the sample/(F/m), e—electron charge/C, *L*—thickness of the sample/m; k—Boltzmann constant/(J/K); *T*—temperature/K; *v*—carrier escape frequency/Hz; *t*—time/s, *V*(*t*)—surface potential/V.

**Figure 15:**
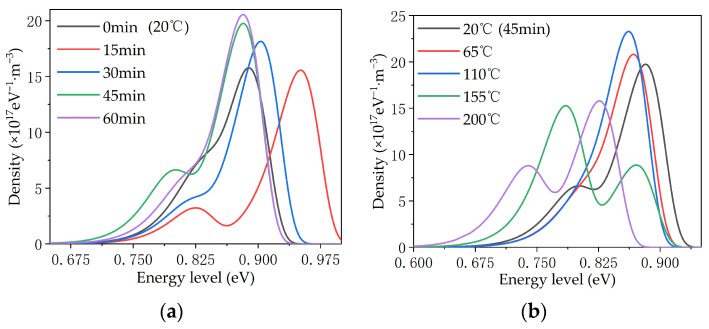
Trap distribution characteristics of the PTFE. (**a**) Different modification times at 20 °C; (**b**) different temperatures at 45 min.

The trap distribution characteristics are shown in Figure 15. According to the different energy levels of traps, they usually divide into shallow traps and deep traps. In the figure, under the condition of 20 °C, the curves of the samples of 15 and 45 min show obvious double peaks, among which the trap densities of samples at 45 min are higher than those of samples at 15 min, on the whole. The trap energy level of the 45 min sample was lower than that of the 15 min sample, while the curves of samples at 0, 30 and 60 min did not show double peaks. However, an obvious transition still took place at the lower energy level, indicating that there are a certain number of shallow traps. For samples at 45 min, the show obvious double peaks at 20, 155, and 200 °C. The deep traps were more numerous than shallow traps at 20 and 200 °C, and this reversed at 155 °C. On the whole, the energy level and density of deep traps did not change much in most tests, but the density of shallow traps changed greatly, as is shown in the curve by the only transition to double peaks. 

According to the Secondary Electron Emission Avalanche (SEEA), electric field distortion most easily occurs at the three points of the cathode electrode, the PTFE material, and the air environment of the surface discharge and aging device, where the first electrons are generated with the increase in voltage. These collide with gas molecules or insulating materials and produce secondary electrons. This process is repeated to form an electron avalanche, which eventually triggers a flashover. The surface discharge and flashover characteristics are closely related to the trap distribution because of the charge capture effect of the surface trap. The accumulated electric charge in the trap causes the electric field here to distort, making discharge more likely to occur, thereby reducing the surface discharge and flashover voltage of the material.

By comparing the results of different modification times shown in Figure 14a and Figure 15a, it can be seen that the surface charge decay rates of the samples at 15, 30, and 45 min gradually increased, and the shallow trap density on the surface also gradually increased, indicating that the increase in the density of shallow traps sped up the surface charge decay. The surface charge decay rates of 45 and 60 min samples were slightly different before 1000 s, and basically the same after 1000 s. The distribution of their surface traps was slightly different before 0.84 eV, and basically the same after 0.84 eV. It can be seen that the charge binding abilities of traps with different energy levels are different, and charges captured by shallower traps dissipated first, while charges captured by deeper traps dissipated later [30]. The surface charge decay rate of the 0 min sample was the fastest, due to the large initial charge accumulation and the influence of the microstructure (except the trap). On the one hand, in the charge injection process, when the electrode was in contact with the PTFE, the Fermi level matching process between the metal electrode and the PTFE occurred, forming a contact barrier, and charge with an energy level higher than the contact barrier could be injected into the PTFE. The contact barrier was mainly determined by the band gap and Fermi level between the electrode and the PTFE. After sputtering the TiO_2_ layer, an additional TiO_2_ band gap was added between the electrode and the PTFE surface, which increased the contact barrier, making charge injection more difficult, and eventually the charge in the shallow trap shielded part of the deep trap [31,32]. On the other hand, the interaction zone between the TiO_2_ particles and the PTFE base introduced a shallower band, which helped form a conductive path, thus accelerating the decay of surface potential [32,33]. These two factors worked together to determine the PTFE material’s behavior as the shallow trap density increased, thus speeding up the dissipation rate of captured electric charges in the trap and inhibiting the electric field distortion caused by the accumulation of electric charges at a fixed position, thus making it more difficult to discharge. The surface discharge and flashover voltage of the PTFE were increased, and finally, the insulation and anti-aging properties of the PTFE were improved.

By comparing the results for different temperature conditions shown in Figure 14b and Figure 15b, it can be seen that for samples with the same modification times, the surface trap energy level decreased as a whole with the increase in temperature, which caused the captured charge to dissipate faster, thus accelerating the surface charge decay. Based on the SEM results shown above, it can be concluded that the effect of temperatures on the performance of modified PTFE can be divided into two aspects: On the one hand, the increase in temperature destroyed the TiO_2_ layer on the surface of the PTFE to a certain extent, resulting in more gully and loss, and weakening the regulating effect of TiO_2_ on the performance of the PTFE. On the other hand, the increase in temperature increased the kinetic energy of the electric charge, causing the captured charge to dissipate faster, but the fast movement of the sample surface charge also sped up the process of SEEA. The above two aspects worked together to reduce the surface discharge and flashover voltage of the material, which ultimately deteriorated the insulation and anti-electric-aging ability of the PTFE. However, compared with unmodified samples, magnetron sputtering TiO_2_ inhibited the deterioration of the PTFE insulation and anti-electric-aging properties at high temperatures to a certain extent, making PTFE materials more suitable for the aviation environment.

## 5. Conclusions

In this paper, the surface insulation and electrothermal aging characteristics of PTFE modified by magnetron sputtering were tested, and the laws of influence of different temperatures and sputtering times were compared and analyzed. Finally, the surface insulation properties of the PTFE were improved. The conclusions are as follows:

1. The insulation performance of the PTFE was affected by temperature. Higher temperatures were shown to significantly advance a partial discharge, while TiO_2_ modification by magnetron sputtering regulated the water contact angle, electrical conductivity, insulation, and anti-electrothermal-aging properties of the sample, which improved the adaptability of the PTFE to the aviation environment;
2. In the electric aging test, the degree of damage of the surface of the sample at 45 min was the lowest. This is because, after modification by sputtering, the discharge voltage increased, the leakage current decreased, the strength of impact of high-energy particles and the heat accumulation damage on the surface were weakened, and the anti-electric-aging ability reached it optimum at that time;
3. By decreasing the trap energy level on the surface of the insulating material and increasing the density of the shallower trap, sputtering modification sped up the electric charge transport and dissipation on the insulating material, inhibited the surface charge accumulation, and then improved the surface insulation performance and anti-aging ability of the insulating material.

## Data Availability

The data presented in this study are available on request from the corresponding author.
